# Association between Human Leukocyte Antigen and End-Stage Renal Disease in Patients from Transylvania, Romania

**DOI:** 10.3390/ijms241713383

**Published:** 2023-08-29

**Authors:** Luminita-Ioana Iancu Loga, Lucia Dican, Alin Dan Chiorean, Vlad Florin Chelaru, Florin Ioan Elec, Cristina Sorina Catana, Monica Mihaela Marta, Roxana Liana Lucaciu, Adriana Corina Hangan, Cosmina Ioana Bondor, Mihaela Laura Vica, Horea Vladi Matei

**Affiliations:** 1 Department of Cellular and Molecular Biology, Faculty of Medicine, “Iuliu-Hațieganu” University of Medicine and Pharmacy, 400012 Cluj-Napoca, Romania; luminisloga@yahoo.com (L.-I.I.L.); chiorean_alin_dan@yahoo.com (A.D.C.); mvica@umfcluj.ro (M.L.V.); hmatei@umfcluj.ro (H.V.M.); 2 Clinical Institute of Urology and Renal Transplantation, 400000 Cluj-Napoca, Romania; ioan.elec@gmail.com; 3 Department of Medical Biochemistry, Faculty of Medicine, “Iuliu-Hațieganu” University of Medicine and Pharmacy, 400012 Cluj-Napoca, Romania; ccatana0128@gmail.com; 4 Department of Medical Education, Faculty of Medicine, “Iuliu-Hațieganu” University of Medicine and Pharmacy, 400012 Cluj-Napoca, Romania; vladflorinchelaru@gmail.com (V.F.C.); mmarta@umfcluj.ro (M.M.M.); 5 Department of Urology, Faculty of Medicine, “Iuliu-Hațieganu” University of Medicine and Pharmacy, 400012 Cluj-Napoca, Romania; 6 Department of Pharmaceutical Biochemistry and Clinical Laboratory, Faculty of Pharmacy, “Iuliu-Hațieganu” University of Medicine and Pharmacy, 400012 Cluj-Napoca, Romania; roxanaluc@yahoo.com; 7 Department of Inorganic Chemistry, Faculty of Pharmacy, “Iuliu-Hațieganu” University of Medicine and Pharmacy, 400012 Cluj-Napoca, Romania; acomsa6@yahoo.com; 8 Department of Medical Informatics and Biostatistics, Faculty of Medicine, “Iuliu-Hațieganu” University of Medicine and Pharmacy, 400012 Cluj-Napoca, Romania; cbondor@umfcluj.ro

**Keywords:** human leukocyte antigen, allele frequency, haplotype frequency, end-stage renal disease

## Abstract

End-stage renal disease (ESRD) is the final stage of chronic kidney disease. This study explored the association between human leukocyte antigen (HLA) and ESRD. The interaction between genetic and environmental factors may also play a role in the development of ESRD. The study included 2392 ESRD patients who were awaiting renal transplantation. Blood samples were genotyped by SSOP and SSP-PCR methods. Multivariate logistic regression analysis showed that HLA-A*11 (*p* = 0.027), HLA-A*34 (*p* = 0.017), HLA-A*69 (*p* = 0.012), HLA-B*41 (*p* < 0.001), HLA-B*50 (*p* = 0.004), HLA-DRB1*10 (*p* = 0.027), and HLA-DRB1*14 (*p* = 0.004) were positively associated with ESRD (OR > 1); HLA-DRB1*07 (*p* < 0.001), HLA-DRB1*08 (*p* = 0.005), and HLA-DRB1*13 (*p* < 0.001) were protective against ESRD (OR < 1); and the three-locus haplotype HLA-A*02–B*41–DRB1*03, containing one susceptible allele, was strongly associated with ESRD (*p* < 0.001, OR = 3.15). In conclusion, this retrospective analysis of HLA typing in patients with ESRD of various etiologies suggests that molecular data on the HLA polymorphism should be collected in order to identify high-risk ESRD patients and to improve graft survival after kidney transplantation.

## 1. Introduction

Numerous genetic and environmental risk factors contribute to kidney disease and complicate the identification of underlying pathophysiological mechanisms [1]. Chronic kidney disease (CKD) is a progressive illness that affects more than 10% of the global population, being the 16th most common reason for years of life lost worldwide [2]. In Romania, the prevalence of CKD is more than ten times higher in hypertensive adults compared with the normal population [3]. However, the prevalence of CKD in Romania was found to be around 7%, which is lower than in other European countries [4]. 

The interaction between genetic and environmental factors may play a role in the development of ESRD. For example, a person with a genetic predisposition to autoimmune disease may be more susceptible to kidney damage from environmental toxins than someone without the genetic predisposition. 

Human leukocyte antigen (HLA) allele polymorphisms are associated with a variety of kidney diseases [5,6]. Human HLA genes, which are found on chromosome 6, are responsible for encoding major histocompatibility complex proteins (6p21). The success of transplantation is determined by the cellular and humoral immune response, which both depend on HLA. The HLA system also contributes to immune response induction, control, and T-cell repertoire selection [7,8]. 

HLA genes and some of the loci encoding them are some of the most polymorphic genes in the human genome. Different subsets of peptides encoded by HLA molecules and their binding specificities are important for understanding the differences in the immune response between individuals [9]. The IPD-IMGT/HLA database is a repository for the variant sequences of HLA alleles. In April 2022, the IPD-IMGT/HLA database reported 33.490 HLA alleles [10].

Advanced molecular techniques, which allow HLA typing, provide an important amount of genetic information regarding the hereditary transmission of some pathologies and donor–recipient compatibility in organ transplantation [11,12]. Kidney transplantation is the optimal therapeutic strategy for end-stage renal failure [12]. The HLA system plays a part in the host immune defense mechanism, while HLA matching is a major challenge for graft rejection in transplantation [13]. HLA genes are divided into two classes: I and II. HLA class I includes three main loci: HLA-A, HLA-B, and HLA-C. HLA class II includes HLA-DP, HLA-DQ, and HLA-DR. The loci HLA-A, HLA-B, and HLA-DR are the most important in kidney transplantation [14]. HLA genes are very polymorphic, which predicts greater susceptibility to several diseases compared with all the other known genes. The association between HLA alleles and renal disorders has already been described [15]. 

In recent years, the association between HLA alleles and ESRD has been suggested, as several HLA class I and class II alleles were found to either be protective or risk factors for ESRD in various studies worldwide. Therefore, genetic association studies conducted in different populations are essential for providing more evidence on globally susceptible HLA alleles and for identifying new alleles associated with particular ESRD patients in a country or area [16]. Although many ESRD-associated HLA alleles have been reported, the results of various studies are inconsistent [17]. This might be caused by a limited sample size or by the existence of specific susceptible alleles or variations among different ethnic groups or races [18].

This study aimed to perform a retrospective analysis of HLA typing in patients diagnosed with ESRD of various etiologies, as well as to establish the HLA frequency and its association with the risk of renal pathology. Representative sample data for our geographic region were retrospectively analyzed to study the genetic loci of HLA class I (HLA-A, HLA-B) and class II (HLA-DRB1) in the Transylvanian population and in ESRD patients wait-listed for renal transplantation. We also described the relationship between HLA and ESRD to establish which HLA alleles predispose to or protect against ESRD. 

## 2. Results

### 2.1. General Characteristics of the Study Population 

HLA patients with ESRD were divided into 22 groups according to the primary disease leading to ESRD, the most common being chronic glomerulonephritis (GN) (Table 1).

### 2.2. HLA-A, -B, -DRB1 Allele Frequencies: Patients vs. Controls

We identified 64 different alleles, including 19 HLA-A alleles, 32 HLA-B alleles, and 13 HLA-DRB1 alleles. The HLA-A, HLA-B, and HLA-DR allele frequencies for the 2392 patients with ESRD and the 3274 controls are summarized in Table 2. 

The most common HLA-A alleles were HLA-A*02, HLA-A*01, HLA-A*24, and HLA-A*03, with a frequency greater than 10% in both groups. HLA-A*74 was found only in the control group, and HLA-A*36 was found only in the patient group. In total, 32 HLA–B alleles were detected in the two groups; of these, the frequency of HLA-B*18 and HLA-B*35 was greater than 10% in both groups. HLA-B*54 was present only in the control group, while HLA-B*70 and HLA-B*78 were present only in the patient group. A total of 13 HLA-DR alleles were detected in the patient group, where the top three alleles were HLA-DR*11, HLA-DR*16, and HLA-DR*03, with a frequency of 21.11%, 10.05%, and 12.19%, respectively (Table 2). 

The following alleles had a statistically significant protective effect (OR < 1): HLA-A*30, HLA-B*38, HLA-B*44, HLA-B*48, HLA-DR*07, HLA-DR*08, HLA-DR*13, while alleles HLA-A*11, HLA-A*34, HLA-A*69, HLA-B*41, HLA-B*50, HLA-B*51, HLA-DRB1*10, HLA-DRB1*11, and HLA-DRB1*14 were found to have a statistically significant risk effect (OR > 1) (Table 3).

When analyzing each allele in homozygote and heterozygote patients, the following alleles, in their homozygous form, were risk factors for renal disease: HLA-A*23 (OR = 11.25, *p* = 0.006), HLA-A*24 (OR = 1.65, *p* = 0.023), HLA-B*08 (OR = 1.94, *p* = 0.021), HLA-B*27 (OR = 5.13, *p* = 0.011), HLA-DRB1*11 (OR = 1.9, *p* = 0.021), HLA-DRB1*14 (OR = 3.05, *p* = 0.019), and HLA-DRB1*16 (OR = 2.17, *p* = 0.001). No HLA allele had a statistically significant protective effect against CKD when in a homozygous form. The results of this analysis, as well as of other analyses, are available in Supplementary File S1.

The logarithmic regression model used to adjust the effect of each allele, taking into consideration the presence (or absence) of other alleles, had an intercept of −0.288 and an accuracy of 59.8%. Compared with the univariate analysis, where individual HLA alleles were assessed independently, the multivariate logistic regression model allowed us to control for potential confounding factors caused by the presence of multiple HLA alleles. As a result, certain alleles that showed significant effects in the univariate analysis, such as HLA-B*38, HLA-B*51, and HLA-DRB1*11, appeared to have no statistically significant effect on the risk of ESRD after adjusting for the effect of other HLA alleles. However, after applying the multivariate logistic regression analysis, we found that HLA-A*11, HLA-A*34, HLA-A*69, HLA-B*41, HLA-B*50, HLA-DRB1*10, and HLA-DRB1*14 exhibited a susceptibility effect to ESRD, indicating their independent association with the disease. In addition, we found that HLA-A*30, HLA-B*44, HLA-B*48, HLA-DRB1*07, HLA-DRB1*08, and HLA-DRB1*13 were protective alleles, indicating that the individuals carrying them had a reduced risk of ESRD (Table 3).

### 2.3. Analysis of HLA-A–B–DRB1 Haplotype Frequencies 

Using the Expectation–Maximization algorithm implemented through the haplo.em function of the haplo.stats package, we generated relative and expected absolute frequencies for all possible haplotypes in the case and control groups. We obtained 1818 unique haplotypes, of which 578 (31.79%) were represented in both groups. We reported data on the first 50 most common haplotypes, out of which 10 were risk haplotypes and 6 were protective haplotypes (Table 4). All the generated haplotypes, as well as their relative and expected absolute frequencies, are available in Supplementary File S2.

We determined the HLA-A–B–DRB1 three-locus haplotypes present in our samples. The haplotypes positively associated with ESRD were A*24–B*18–DRB1*11, A*01–B*40–DRB1*14, A*02–B*35–DRB1*11, A*02–B*27–DRB1*16, A*11–B*35–DRB1*11, A*25–B*18–DRB1*04 A*01–B*35–DRB1*11, A*11–B*35–DR*04, A*02–B*51–DRB1*15, and A*02–B*41–DRB1*03, while A*01–B*08–DRB1*03, A*02–B*18–DRB1*11, A*03–B*35–DRB1*01, A*03–B*07–DRB1*15, A*02–B*44–DRB1*04, and A*26–B*38–DRB1*13 were negatively associated with ESRD. This analysis revealed that HLA-A*24–B*18–DR*11 is the most common risk haplotype for ESRD, with its frequency in the case group being 12.01‰, while the frequency in the control group was 7.76‰, which is significantly lower than the frequency in ESRD patients (*p* < 0.05).

## 3. Discussion

ESRD is a major health concern in Europe. According to data from the European Renal Association–European Dialysis and Transplant Association (ERA-EDTA), the prevalence of ESRD in Europe has been steadily increasing in recent years. 

HLA is thought to be one of several contributing factors to the development and progression of ESRD. The results of the current study illustrate how the expression of ESRD is influenced by HLA class I and II alleles. HLA polymorphisms, which are involved in the control of immunological processes, are related to a high number of renal or systemic diseases [17,19].

There are various aspects related to renal involvement in autoimmunity [20,21]. HLA genes play a critical role in the ability of the immune system to recognize and respond to foreign substances, such as viruses and bacteria [22,23]. In the context of ESRD, dysregulation of the immune system, including aberrant expressions of HLA antigens, has been implicated in the development of kidney damage and inflammation.

HLA types can either predispose to or protect against the development of autoimmune disease via a variety of mechanisms, including changes in HLA expression or stability, antigenic peptide modifications, changes in the peptide-binding register between different HLA molecules, or the expansion of a pathogenic or protective antigen-specific T cell repertoire [20].

Although the immune system is involved in many types of renal diseases, there is no universally accepted definition of the term autoimmune kidney disease [21]. The autoimmune diseases accompanied by autoantibody production are typically associated with HLA class II, while the diseases not accompanied by this phenomenon are more commonly associated with certain HLA class I alleles. 

Glomerulonephritis is usually classified into primary and secondary forms. Secondary glomerulonephritis can be seen in systemic inflammatory diseases such as small vessel vasculitis and systemic lupus erythematosus. The classification of primary glomerulonephritis is debatable and confusing. A major cause of confusion is the poor correlation between histological and clinical findings, which generates considerable overlaps between diseases defined by clinical features and diseases defined by histological features [24]. 

Glomerular, tubular, and vascular structures are targeted and damaged as a consequence of autoimmune processes. Some of the target autoantigens have now been identified in autoimmune diseases where the tissue injury includes the kidney [21]. Such autoimmune diseases are characterized by systemic inflammation leading to target organ dysfunction, including of the kidneys. Sex differences in the incidence and severity of these diseases result from a complex interaction of hormonal, genetic, and epigenetic factors.

In this retrospective study, a total of 19 HLA-A alleles, 32 HLA-B alleles, and 13 HLA-DR alleles were detected in the case and control groups. In the case group, the top three alleles with the highest frequencies in each of the three loci were HLA-A*02 (28.11%), HLA-A*01 (14.19%), HLA-A*24 (11.85%), HLA-B*35 (15.57%), HLA-B*18 (10.79%), HLA-B*51 (10.05%), HLA-DRB1*11 (21.11%), HLA-DRB1*03 (12.19%), and HLA-DRB1*16 (10.05%). HLA frequencies in the control group were concordant with the distribution of HLA allele frequencies in the Romanian population from Transylvania [25]. 

The present study analyzed the HLA-A, HLA-B, and HLA-DRB1 allele frequencies and haplotype distributions in ESRD patients from Transylvania. We found that HLA-A*24–B*18–DRB1*11 (OR = 1.56, *p* = 0.031) showed significantly different distributions between ESRD patients (12.01‰) and controls (7.76‰), thus indicating that they were the most common risk haplotypes for ESRD in our population. On the other hand, HLA-A*02–B*41–DRB1*03 (OR = 3.15, *p* < 0.001), carrying the susceptible allele HLA-B*41, was the most susceptible haplotype for ESRD.

Our results also indicated that alleles HLA-A*11, HLA-A*34, HLA-A*69, HLA-B*41, HLA-B*50, HLA-DRB1*10, and HLA-DRB1*14 were associated with the risk of ESRD. These results are in agreement with those of Noureen et al. [26]. The HLA-A*11 allele was also positively associated with ESRD in a previous study performed by Maruntelu et al. in the Romanian population [27]. However, the results of our study differed from those of Cao et al., who described an association between allele HLA*24 and ESRD [5].

According to the univariate analysis, HLA-B*51 was the most numerous HLA-B allele in our patient group. This result is similar to that reported in Egyptian, Turkish, and Saudi Arabian populations [28]. After applying the multivariate analysis, HLA-B*51 did not appear to be associated with the risk of ESRD in our population. In our study, alleles HLA-B*41 and HLA-B *50 conferred susceptibility to ESRD. A significant susceptibility association was found between ESRD and A*11 in immunoglobulin A nephropathy and Henoch–Schönlein purpura [29]. Moreover, at the DRB1 locus, DRB1*10, DRB1*11, and DRB1*14 emerged as susceptible alleles for ESRD. HLA-DRB1*11 was reported to be a susceptible allele conferring the risk of myeloperoxidase (MPO) antineutrophil cytoplasmic antibody (ANCA)-associated vasculitis (AAV), i.e., MPO-AAV, in a Chinese population [30,31].

In a systematic review of patients with ESRD, Lowe et al. showed that HLA-B*38 had a contrary effect to the one reported by us [32]. In another study, the distribution of HLA genotypes was different in Transylvania compared with other regions in Romania [33]. However, the Hungarian minority population is more numerous in Transylvania compared with the other regions in Romania, and the ethnicity of the population can influence the distribution of HLA haplotypes and their association with ESRD [27].

To our knowledge, this study is the first in northern Romania (Transylvania) to analyze the association of different HLA alleles and haplotypes with ESRD. Allele and haplotype distributions vary in different geographic locations and ethnic groups.

Freedman et al. found an increased frequency of HLA-B*35 in patients with ESRD due to hypertensive renal failure [34]. Associations with HLA-B*35 were also reported by Forsberg and Lowe in patients with malignant hypertension and terminal uremia [32]. Furthermore, the presence of HLA-B*35 was confirmed in a study on white European nephropathy patients with malignant hypertension [35]. Thus, HLA-B*35 could represent a marker for severe hypertension in renal disease. However, we did not find any association between HLA-B*35 and ESRD.

An association between HLA-DRB1*11 and ESRD was reported in Taiwanese, Chinese, and Romanian populations [27,36,37]. Only 10 HLA associations with ESRD were reported in three or more studies (HLA-A*11, B*07, B*08, B*53, DRB1*03, DRB1*04, DRB1*08, DRB1*11, DQB1*02, and DQB1*06), and 6 of these were refuted by another study (the exceptions being HLA-B*53, DRB1*08, DQB1*02, and DQB1*06) [24,38].

Future studies could provide a more comprehensive statistical analysis by taking into consideration clinical characteristics, including age, gender, or even the presence of other comorbidities as independent variables in logistic regression models, despite the difficulty of anonymization, especially in donor groups. Moreover, multicenter studies could provide better information about the risk associated with specific HLA alleles, irrespective of ethnicity or local biases, such as the different prevalence of HLA alleles around the world. In this respect, we acknowledge the need to initiate research collaborations with similar institutions or organizations from Central and Southeastern Europe in order to improve the quality of life of ESRD patients by creating relevant clinical and paraclinical instruments as well as models for predicting the risk of ESRD. This could facilitate personalized transplantation based on genetic risk, thus improving treatment outcomes. 

## 4. Materials and Methods

A total of 5666 individuals were enrolled in this retrospective study between 2013 and 2021. The individuals were divided into two groups: 2392 ESRD patients from the waiting list for renal transplantation at the Clinical Institute of Urology and Renal Transplantation, Cluj-Napoca and 3274 healthy individuals who had registered as stem cell donors under the Romanian Voluntary Bone Marrow Donor Registry (RVBMDR).

The 2392 patients enrolled in our study included 1483 males (61.99%) and 909 females (38.01%), with a mean age of 45.685 ± 12.336 years. The control group included 3274 healthy volunteers, of whom 1853 were male (56.05%) and 1439 female (43.95%), with ages ranging from 18 to 45 years. The control group was not age- and gender-matched with the patient group because age and gender do not influence the HLA frequency profile.

The study was approved by the ethics committees of the Clinical Institute of Urology and Renal Transplantation, Cluj-Napoca and of the “Iuliu Hațieganu” University of Medicine and Pharmacy in Cluj-Napoca (no. 326/01.10.2019). 

The patients were divided into 22 groups according to the etiology of renal disease. Patients who had chronic malignancies were excluded from the study. 

We aimed to study the alleles in the HLA class I region (A, B) and HLA class II region (DR) in order to detect the alleles that confer susceptibility to or protection against ESRD. For HLA typing, Polymerase Chain Reaction (PCR) techniques such as PCR–Sequence-Specific Primer (PCR-SSP) or PCR–Sequence-Specific Oligomer (PCR-SSO) are relatively simple, fast, and automated, providing information at a low resolution. These relatively recently developed methods in the study of DNA profiles through HLA typing are based on the amplification of HLA regions with a high degree of polymorphism, followed by HLA typing by allele or group of alleles.

### 4.1. DNA Extraction

Two mL of peripheral venous blood was collected from patients and controls in Vacutainer anticoagulant tubes with ethylenediaminetetraacetic acid (EDTA) according to the manufacturer’s protocol. DNA was extracted using an innuPREP Blood DNA Mini kit IPC16 (Analytik Jena AG, Berlin, Germany). The DNA concentration was adjusted to 10–30 ng/μL. Nanophotometric readings against a reference Tris buffer were used to quantify the DNA concentration and purity. 

### 4.2. HLA genotyping

The PCR-SSO method was used with the HISTO SPOT A. B. DRB1 kit (Bag Diagnostics GmbH, Lich, Germany). HLA data were analyzed with HISTO MATCH Software (V4.X-03/2020, Bag Diagnostics GmbH, Lich, Germany). Ambiguous HLA typing was retested via PCR-SSP using the HLA A-B-DR SSP Combi Tray (CareDx, Stockholm, Sweden) according to the manufacturer’s instructions. The results were processed with the Helmberg SCORE software version 5.00.41T.

### 4.3. Statistical analysis

The data were organized using Microsoft Excel, Microsoft Office 2019 suite (Microsoft Corp., Redmond, USA) and then analyzed using R 4.2.2 (R Foundation) [39] and RStudio (Posit Software, PBC. Boston, MA, USA) [40]. The following libraries were loaded onto the workspace: stringr [41], readxl [42], xlsx [43], and haplo.stats [44].

For each patient, we computed their case/control status and the number of copies of each allele analyzed. We only analyzed alleles identified in at least one member of each group. We then tested for statistically significant associations between the presence of each HLA allele and the case/control status using either the Chi^2^ test or, if the assumptions were violated (due to small theoretical frequencies), Fisher’s exact test. The association was quantified through the odds ratio (OR) and its 95% confidence interval (CI). 

Additionally, for each allele, we analyzed whether homozygous patients had an increased risk of developing CKD compared with heterozygous patients. We used either the Chi^2^ test or Fisher’s exact test, and we computed the OR and its 95% CI.

Next, to adjust the effect of each allele depending on the presence or absence of other alleles, alleles with *p* < 0.1 for Chi^2^ or Fisher’s exact tests were included in a multivariate logistic regression analysis as independent variables, with the case/control status being the dependent variable. For each HLA allele in the model, we reported the computed OR (the base of the natural logarithm raised to the allele coefficient β), its 95% CI, and its *p* value. For the entire model, we reported the intercept and the model accuracy measured against the data.

Lastly, using the haplo.em function in the haplo.stats package, which is based on the Expectation–Maximization algorithm, we generated relative frequencies for all the haplotypes in both groups. We then computed the expected absolute frequencies by multiplying the relative frequencies of each allele with double the number of cases and controls (due to each patient having two haplotypes). Finally, we quantified the risk or protective effect of each haplotype against renal disease through OR and we tested the statistical significance of the effect through the Chi^2^ or Fisher’s tests. The results were considered statistically significant if *p* < 0.05.

## 5. Conclusions

The present study has provided significant information about the frequency of HLA alleles and haplotypes in Romanian patients with end-stage renal disease awaiting renal transplantation. Our analyses identified that alleles HLA-A*11, HLA-A*34, HLA-A*69, HLA-B*41, HLA-B*50, HLA-DRB1*10, and HLA-DRB1*14 were associated with the risk of developing ESRD. The haplotype HLA-A*02–B*41–DRB1*03, containing one susceptible allele (HLA-B*41), was regarded as the haplotype most susceptible to ESRD. In conclusion, this retrospective analysis of HLA typing in patients with ESRD of various etiologies suggests that molecular data on HLA polymorphisms should be collected in order to identify high-risk ESRD patients and to improve graft survival after kidney transplantation.

## Figures and Tables

**Table 1:** Number of patients according to the etiology of their CKD.

Etiology of CKD	No. of Cases	Percentage (%)
Amyloidosis	7	0.29
Polycystic kidney disease	285	11.91
Acquired cystic kidney disease	30	1.25
Membranoproliferative glomerulonephritis	33	1.38
Segmental focal glomerulosclerosis	44	1.84
Chronic glomerulonephritis	774	32.36
Rapidly progressive glomerulonephritis	13	0.54
Renal failure of unspecified cause	290	12.12
Kidney stones	140	5.85
Systemic lupus erythematosus	22	0.92
Alport hereditary nephropathy	22	0. 92
Fabry hereditary nephropathy	5	0.21
Tubulointerstitial nephropathy	149	6.23
IgA nephropathy	99	4.14
Reflux nephropathy	6	0.25
Type 1 diabetic nephropathy	118	4.93
Type 2 diabetic nephropathy	239	9.99
Tubulointerstitial nephritis	19	0.79
Vascular nephropathy	67	2.8
Poststreptococcal nephropathy	21	0.88
Hemolytic uremic syndrome	6	0.25
Total	2392	

**Table 2:** Frequency of HLA-A, HLA-B, and HLA-DR alleles in ESRD patients and healthy control donors and univariate analysis of HLA-A, -B, -DRB1 locus in all cases vs. all controls.

HLA Allele	Number of Allele Copies in ESRD Patients (% of 2n)	Number of Allele Copies in Healthy Controls (% of 2n)	OR (CI 95%)	*p* Value	Effect
A*01	679 (14.19)	924 (14.11)	1.01 (0.9–1.12)	0.902	NS
A*02	1345 (28.11)	1791 (27.35)	1.04 (0.96–1.13)	0.37	NS
A*03	493 (10.31)	714 (10.9)	0.94 (0.83–1.06)	0.307	NS
A*11	430 (8.99)	487 (7.44)	1.23 (1.07–1.41)	0.003	risk
A*23	134 (2.8)	168 (2.57)	1.09 (0.87–1.38)	0.442	NS
A*24	567 (11.85)	763 (11.65)	1.02 (0.91–1.14)	0.744	NS
A*25	158 (3.3)	204 (3.12)	1.06 (0.86–1.31)	0.576	NS
A*26	226 (4.72)	324 (4.95)	0.95 (0.8–1.13)	0.584	NS
A*29	84 (1.76)	113 (1.73)	1.02 (0.77–1.35)	0.942	NS
A*30	71 (1.48)	182 (2.78)	0.53 (0.4–0.69)	<0.001	protective
A*31	113 (2.36)	161 (2.46)	0.96 (0.75–1.22)	0.741	NS
A*32	202 (4.22)	296 (4.52)	0.93 (0.78–1.12)	0.445	NS
A*33	82 (1.71)	139 (2.12)	0.8 (0.61–1.06)	0.13	NS
A*34	9 (0.19)	2 (0.03)	6.17 (1.33–28.57)	0.011	risk
A*36	2 (0.04)	0 (0)			
A*66	34 (0.71)	49 (0.75)	0.95 (0.61–1.47)	0.911	NS
A*68	134 (2.8)	208 (3.18)	0.88 (0.7–1.09)	0.248	NS
A*69	21 (0.44)	12 (0.18)	2.4 (1.18–4.89)	0.02	risk
A*74	0 (0)	11 (0.17)			
B*07	271 (5.66)	396 (6.05)	0.93 (0.8–1.09)	0.392	NS
B*08	375 (7.84)	556 (8.49)	0.92 (0.8–1.05)	0.212	NS
B*13	152 (3.18)	232 (3.54)	0.89 (0.73–1.1)	0.288	NS
B*14	102 (2.13)	172 (2.63)	0.81 (0.63–1.03)	0.09	NS
B*15	188 (3.93)	269 (4.11)	0.95 (0.79–1.15)	0.634	NS
B*18	516 (10.79)	700 (10.69)	1.01 (0.9–1.14)	0.871	NS
B*27	242 (5.06)	314 (4.8)	1.06 (0.89–1.26)	0.522	NS
B*35	745 (15.57)	951 (14.52)	1.09 (0.98–1.2)	0.122	NS
B*37	58 (1.21)	71 (1.08)	1.12 (0.79–1.59)	0.531	NS
B*38	162 (3.39)	282 (4.31)	0.78 (0.64–0.95)	0.013	protective
B*39	142 (2.97)	159 (2.43)	1.23 (0.98–1.55)	0.077	NS
B*40	260 (5.43)	321 (4.9)	1.11 (0.94–1.32)	0.204	NS
B*41	122 (2.55)	96 (1.47)	1.76 (1.34–2.3)	<0.001	risk
B*42	4 (0.08)	1 (0.02)	5.48 (0.61–49.03)	0.17	NS
B*44	380 (7.94)	662 (10.11)	0.77 (0.67–0.88)	<0.001	protective
B*45	12 (0.25)	19 (0.29)	0.86 (0.42–1.78)	0.721	NS
B*46	1 (0.02)	4 (0.06)	0.34 (0.04–3.06)	0.405	NS
B*47	37 (0.77)	34 (0.52)	1.49 (0.94–2.38)	0.093	NS
B*48	4 (0.08)	20 (0.31)	0.27 (0.09–0.8)	0.012	protective
B*49	102 (2.13)	113 (1.73)	1.24 (0.95–1.63)	0.125	NS
B*50	68 (1.42)	57 (0.87)	1.64 (1.15–2.34)	0.006	risk
B*51	481 (10.05)	577 (8.81)	1.16 (1.02–1.31)	0.025	risk
B*52	89 (1.86)	144 (2.2)	0.84 (0.65–1.1)	0.228	NS
B*53	8 (0.17)	15 (0.23)	0.73 (0.31–1.72)	0.532	NS
B*54	0 (0)	2 (0.03)			
B*55	87 (1.82)	126 (1.92)	0.94 (0.72–1.24)	0.726	NS
B*56	45 (0.94)	56 (0.86)	1.1 (0.74–1.63)	0.686	NS
B*57	82 (1.71)	125 (1.91)	0.9 (0.68–1.19)	0.478	NS
B*58	43 (0.9)	73 (1.11)	0.8 (0.55–1.17)	0.299	NS
B*70	2 (0.04)	0 (0)			
B*73	2 (0.04)	1 (0.02)	2.74 (0.25–30.21)	0.577	NS
B*78	2 (0.04)	0 (0)			
DRB1*01	428 (8.95)	640 (9.77)	0.91 (0.8–1.03)	0.136	NS
DRB1*03	583 (12.19)	724 (11.06)	1.12 (0.99–1.25)	0.063	NS
DRB1*04	475 (9.93)	585 (8.93)	1.12 (0.99–1.28)	0.072	NS
DRB1*07	397 (8.3)	702 (10.72)	0.75 (0.66–0.86)	<0.001	protective
DRB1*08	80 (1.67)	154 (2.35)	0.71 (0.54–0.93)	0.013	protective
DRB1*09	19 (0.4)	17 (0.26)	1.53 (0.8–2.95)	0.237	NS
DRB1*10	87 (1.82)	78 (1.19)	1.54 (1.13–2.09)	0.007	risk
DRB1*11	1010 (21.11)	1232 (18.81)	1.15 (1.05–1.27)	0.002	risk
DRB1*12	85 (1.78)	111 (1.7)	1.05 (0.79–1.39)	0.771	NS
DRB1*13	425 (8.88)	742 (11.33)	0.76 (0.67–0.86)	<0.001	protective
DRB1*14	288 (6.02)	285 (4.35)	1.41 (1.19–1.67)	<0.001	risk
DRB1*15	426 (8.9)	594 (9.07)	0.98 (0.86–1.12)	0.759	NS
DRB1*16	481 (10.05)	684 (10.45)	0.96 (0.85–1.08)	0.498	NS

HLA: human leukocyte antigen; OR: odds ratio; CI: confidence interval; *p* values were calculated by Fisher’s exact test; NS: not significant.

**Table 3:** Multivariate logistic regression analysis in cases/controls as dependent variables and HLA-A, HLA-B, and HLA-DRB1 as independent variables.

HLA Allele	OR (CI 95%)	*p* Value	Effect
A*11	1.18 (1.02–1.37)	0.027	risk
A*30	0.56 (0.42–0.74)	<0.001	protective
A*34	6.59 (1.68–43.52)	0.017	risk
A*69	2.52 (1.24–5.35)	0.012	risk
B*14	0.84 (0.65–1.09)	0.203	NS
B*38	0.84 (0.68–1.04)	0.114	NS
B*39	1.24 (0.97–1.57)	0.083	NS
B*41	1.91 (1.45–2.54)	<0.001	risk
B*44	0.76 (0.65–0.87)	<0.001	protective
B*47	1.49 (0.92–2.41)	0.105	NS
B*48	0.27 (0.08–0.73)	0.018	protective
B*50	1.71 (1.18–2.48)	0.004	risk
B*51	1.1 (0.95–1.26)	0.206	NS
DRB1*03	1.05 (0.91–1.21)	0.483	NS
DRB1*04	1.08 (0.93–1.25)	0.297	NS
DRB1*07	0.77 (0.66–0.9)	<0.001	protective
DRB1*08	0.66 (0.49–0.88)	0.005	protective
DRB1*10	1.44 (1.04–1.99)	0.027	risk
DRB1*11	1.09 (0.96–1.23)	0.188	NS
DRB1*13	0.71 (0.61–0.83)	<0.001	protective
DRB1*14	1.31 (1.09–1.58)	0.004	risk

HLA: human leukocyte antigen; OR: odds ratio; CI: confidence interval; *p* values were calculated by Fisher’s exact test; NS: not significant.

**Table 4:** Three-locus haplotypes with significant risk/protective effects on CKD development.

Haplotype	Frequency in Cases (‰)	Frequency in Controls (‰)	Total Frequency (‰)	OR (CI 95%)	*p* Value	Effect
A*01–B*08–DRB1*03	39.9	50.49	92.03	0.78 (0.65–0.94)	0.008	protective
A*02–B*18–DRB1*11	21.82	31.12	54.38	0.69 (0.55–0.88)	0.003	protective
A*03–B*35–DRB1*01	8.82	13.02	22.5	0.67 (0.47–0.98)	0.038	protective
A*24–B*35–DRB1*11	9.53	10.59	20.28	0.9 (0.62–1.31)	0.704	NS
A*02–B*51–DRB1*16	9.3	10.52	20.01	0.88 (0.6–1.29)	0.504	NS
A*02–B*44–DRB1*16	7.49	11.22	19.29	0.67 (0.45–0.99)	0.052	NS
A*24–B*18–DRB1*11	12.01	7.76	19.11	1.56 (1.06–2.27)	0.031	risk
A*03–B*07–DRB1*15	6.22	10.16	16.99	0.61 (0.39–0.94)	0.023	protective
A*02–B*13–DRB1*07	7.43	8.56	16.16	0.87 (0.57–1.32)	0.597	NS
A*02–B*51–DRB1*11	9.32	7.16	16.14	1.31 (0.86–1.97)	0.204	NS
A*01–B*40–DRB1*14	11.78	5.12	15.86	2.32 (1.51–3.56)	<0.001	risk
A*11–B*35–DRB1*01	6.79	8.75	15.85	0.77 (0.5–1.19)	0.238	NS
A*23–B*44–DRB1*07	6.82	8.35	15.42	0.82 (0.53–1.26)	0.388	NS
A*02–B*35–DRB1*11	11.28	4.89	15.17	2.32 (1.5–3.6)	<0.001	risk
A*02–B*07–DRB1*15	6.38	7.9	14.51	0.81 (0.51–1.26)	0.435	NS
A*02–B*27–DRB1*16	9.43	5.43	14.24	1.74 (1.12–2.71)	0.017	risk
A*11–B*35–DRB1*11	8.71	5	13.13	1.75 (1.1–2.77)	0.019	risk
A*02–B*27–DRB1*01	4.64	6.66	11.62	0.69 (0.42–1.16)	0.169	NS
A*25–B*18–DRB1*15	5.1	6.19	11.45	0.82 (0.5–1.36)	0.45	NS
A*01–B*35–DRB1*11	7.69	4.24	11.4	1.82 (1.11–2.98)	0.023	risk
A*02–B*15–DRB1*04	4.01	6.12	10.46	0.65 (0.38–1.13)	0.146	NS
A*33–B*14–DRB1*01	4.35	5.73	10.3	0.76 (0.44–1.3)	0.355	NS
A*02–B*44–DRB1*11	4.12	5.85	10.24	0.7 (0.41–1.21)	0.286	NS
A*24–B*08–DRB1*03	6.5	4.04	10.16	1.61 (0.96–2.71)	0.08	NS
A*02–B*18–DRB1*16	4.96	5.09	10.07	0.97 (0.57–1.65)	1	NS
A*30–B*13–DRB1*07	4.46	5.41	10.02	0.82 (0.48–1.41)	0.501	NS
A*02–B*08–DRB1*03	5.74	4.42	9.95	1.3 (0.77–2.2)	0.416	NS
A*25–B*18–DRB1*04	7.35	3.21	9.92	2.3 (1.34–3.95)	0.003	risk
A*26–B*38–DRB1*04	3.42	5.86	9.66	0.58 (0.33–1.04)	0.072	NS
A*11–B*35–DRB1*04	7.62	2.66	9.52	2.88 (1.62–5.09)	<0.001	risk
A*02–B*35–DRB1*14	5.36	4.26	9.44	1.26 (0.74–2.16)	0.409	NS
A*11–B*18–DRB1*11	4.39	4.94	9.42	0.89 (0.51–1.54)	0.781	NS
A*32–B*35–DRB1*11	4.16	4.85	9.12	0.86 (0.49–1.5)	0.673	NS
A*02–B*44–DRB1*04	1.69	6.37	8.78	0.26 (0.12–0.56)	<0.001	protective
A*26–B*38–DRB1*13	2.66	5.43	8.52	0.49 (0.26–0.93)	0.029	protective
A*11–B*52–DRB1*15	4.55	3.91	8.37	1.17 (0.66–2.07)	0.661	NS
A*03–B*18–DRB1*16	3.3	4.81	8.34	0.68 (0.37–1.25)	0.301	NS
A*02–B*35–DRB1*01	4	4.27	8.31	0.94 (0.52–1.68)	0.883	NS
A*68–B*18–DRB1*11	4.6	3.73	8.19	1.23 (0.69–2.2)	0.457	NS
A*03–B*51–DRB1*11	4.22	3.77	7.92	1.12 (0.62–2.02)	0.765	NS
A*24–B*38–DRB1*13	2.62	4.83	7.79	0.54 (0.28–1.04)	0.072	NS
A*02–B*51–DRB1*15	8.57	0.28	7.56	30.75 (7.02–134.71)	<0.001	risk
A*32–B*40–DRB1*16	3.13	4.03	7.31	0.78 (0.41–1.46)	0.528	NS
A*24–B*35–DRB1*04	3.52	3.74	7.29	0.94 (0.51–1.76)	1	NS
A*02–B*41–DRB1*03	6.01	1.92	7.29	3.15 (1.62–6.13)	<0.001	risk
A*24–B*07–DRB1*15	3.74	3.51	7.22	1.07 (0.57–1.98)	0.875	NS
A*02–B*52–DRB1*15	4.79	2.59	7.03	1.85 (0.99–3.48)	0.055	NS
A*01–B*57–DRB1*07	3.88	3.18	6.95	1.22 (0.65–2.29)	0.524	NS
A*29–B*44–DRB1*07	3.68	3.25	6.86	1.13 (0.6–2.13)	0.629	NS
A*02–B*44–DRB1*13	2.36	4.13	6.77	0.57 (0.28–1.14)	0.103	NS

Haplotype: set of loci (HLA-A, HLA-B, HLA-DRB1); OR: odds ratio; CI: confidence interval; *p* values were calculated by Fisher’s exact test; NS: not significant.

## Data Availability

The data presented in this study are available within the article.

## Supplementary Materials

The following supporting information can be downloaded at: https://www.mdpi.com/article/10.3390/ijms241713383/s1.
